# Pathophysiology of writer’s cramp: an exploratory study on task-specificity and non-motor symptoms using an extended fine-motor testing battery

**DOI:** 10.1186/s40734-017-0060-4

**Published:** 2017-08-08

**Authors:** Ali Amouzandeh, Michael Grossbach, Joachim Hermsdörfer, Eckart Altenmüller

**Affiliations:** 1 Institute of Music Physiology and Musicians’ Medicine (IMMM), University of Music, Drama and Media, Emmichplatz 1, 30175 Hannover, Germany; 2 Institute of Human Movement Science, Department of Sport and Health Sciences, Technical University of Munich, Munich, Germany

## Abstract

**Background:**

Writer’s cramp (WC) is a task-specific focal dystonia which manifests itself as abnormal postures interfering with motor performance. As the spread of motor symptoms remains controversial and non-motor symptoms are widely discussed, in this exploratory study, we explore the pathophysiology of WC, focusing on task-specificity and the psychological profiles of WC patients.

**Methods:**

In 14 right-handed WC patients and matched controls, we assessed motor control by applying motor performance tests (Vienna Test Series), as well as using writing analysis and grip-force measurements. Moreover, detailed psychological factors were assessed. Classification trees were used to distinguish patients from controls.

**Results:**

The total duration of writing and the vertical writing frequency of the pen are the most important variables to split the data set successfully into patients and controls. No other variables concerning motor performance tests, grip-force measurements or psychological factors correctly separated patients and controls.

**Conclusions:**

Only variables from the writing tasks successfully separated patients and controls, indicating a strong task-specificity of WC in our patient group. Future research should be performed with larger samples of untreated WC patients in early stages of impairment, without any secondary motor disturbances, to verify our findings.

## Background

Writer’s Cramp (WC) is a task-specific movement disorder that manifests itself as abnormal postures and unwanted muscle spasms that interfere with motor performance while writing [1]. According to the new classification, WC is considered a sporadic focal dystonia (FD) with late adult onset between the ages of 30 and 50 years [2].

One symptom of WC, typically during the initial stage, is a tight grip on the pen. Hand–wrist flexors are more commonly involved than extensors, though hyperextension of the distal phalanges or even the fingers may occur [3]. Slowly, handwriting becomes less legible. About half of the patients with simple cramps progress to having dystonia with other activities. Remissions are uncommon, and symptoms can progress to the other hand in about 5% of cases [4].

Symptoms appear at a mean age of 38 years [3]. Generally, FD of the limb is rare, and prevalence has been estimated in a more recent meta-analysis to be 15 per 100,000 people [5]. The prevalence rate of WC was reported to be 6.9 per 100,000 persons, whereas the incidence was 0.27 per 100,000 in one year [6].

It is still under debate whether WC is task-specific or not. Task-specificity in general remains a fascinating topic in focal dystonia, and it is still not completely understood (see Pirio Richardson et al. 2017 for an actual discussion [7]). Brain imaging studies revealed that the connectivity between the parietal and premotor areas was weaker. It appears that a specific parietal-premotor pathway is malfunctioning in WC [8]. In clinical practice, an initial classification divided the patients into two groups, those with simple and those with dystonic WC, on the basis of the absence or presence of dystonia while performing other tasks [9]. However, this simple classification seems inappropriate, as there may be transitions from highly specific deficits, which only affect the writing of specific letters [10], to simple and then to dystonic WC, and eventually to multifocal dystonias. Moreover, even patients with simple WC frequently report discomfort in other daily activities, like typing on a computer keyboard [11] or using a spoon [12]. Marsden et al. noted frequent association of other features of segmental and generalized dystonia in patients with dystonic WC over 30 years ago [7] and, nowadays, even the spread of motor symptoms to the opposite hand are reported [13]. Secondary motor disturbances with reduced range of motion in other task than writing have also been described in patients suffering from WC [14]. These were related to the severity and duration of the disorder and explained by biomechanical abnormalities of the hand, possibly as a consequence of a combination of innate factors and long-term effects of treatment with botulinum toxin. Indeed, in other focal dystonias, biomechanical abnormalities might contribute to the development. Wilson et al. [15] showed increased stiffness and reduced range of motion in fingers in 10 out of 14 musicians suffering from musician’s dystonia (MD), interestingly also in the unaffected hand. This was also impressively demonstrated in a guitarist with MD [16]. Such biomechanical abnormalities might affect other fine motor tasks, if they are similar to the dystonic task. In keeping with this, such secondary motor disturbances were present in 53% of MD patients [17] when movements were very similar to the main affected task; for example, playing piano and typing on a computer keyboard. However, in a previous study applying the same methodology as in the present paper, Hofmann and colleagues (2015) could not find such spread of symptoms [18]. Similarly, Schneider et al. [19] investigated grip force in patients with WC and did not find a spread of symptoms to other sensorimotor tasks [19].

The primary goal of the present study was to clarify whether a spread of symptoms to other fine motor task could be demonstrated. We applied an extensive fine motor test battery, targeted at writing movements, other controlled guided fine motor movements, ballistic targeting movements and grip force. The test battery applied is the largest one represented in the literature and exceeds the one applied by Schneider and colleagues [19].

An additional goal of this study was related to assess psychological factors related to the triggering of WC. In a recent study on MD, Ioannou and Altenmüller demonstrated that 56% of musicians with dystonia were suffering from psychological symptoms, such as increased trait anxiety and obsessive-compulsive behavior [20]. These premorbid psychological factors seem to play a role in triggering task-specific dystonia, since, on average, symptoms of dystonia occurred 10 years earlier in musicians with psychological issues as opposed to MD patients with no elevated levels of stress and anxiety [21]. Here, we wanted to address the question, whether anxiety, perfectionism and other psychological symptoms might also contribute to triggering WC.

## Methods

A total of 15 WC patients participated in the study. As we intended to investigate a homogeneous population, one of the patients was excluded from further analyses because of being left-handed, according to the Edinburgh Handedness Inventory [22]. All patients (7 females, 8 males) had been diagnosed with WC by a movement disorders specialist (senior author EA) and were recruited from the outpatient clinic at the Institute of Music Physiology and Musicians’ Medicine at the Hanover University of Music, Drama and Media. The institute is registered with the German health board, and offers treatment for non-musician patients suffering from task-specific movement disorders. None of the patients in the present study were professional musicians. Patients did not suffer from any other neurological deficit; in particular, musicians’ dystonia was excluded. The mean age of the subjects was 47.20 years (SD: 12.99; range: 26.67 to 69.67). Of 14 patients, 13 were affected in the right (dominant) hand or arm, while one was suffering from symptoms in both hands or arms, though mainly in the right hand or arm. No patient had symptoms in the left hand or arm exclusively. Eight of the 14 patients were amateur musicians, who had played their instrument for an average of 19.83 years (SD: 14.39; range: 8 to 40). Even though some discomfort, e.g. perceived tension after prolonged playing was reported by 5 of the 8 amateur musicians, patients did not suffer from MD with involuntary flexion or extension of fingers etc. The duration of WC amounted to a mean of 9.52 years (SD: 5.48; range: 0.5 to 19).

Ten patients had been treated with Botulinum Toxin (BT), partly combined with trihexyphenidyl (THX) or an additional retraining/physiotherapy to reduce symptoms of WC. In all cases, the last treatment of BT had taken place more than 6 months before the study. As several studies have shown that the effect of BT lasts about 12 weeks [23], a clinical effect of the medication on the results can most likely be excluded. Three patients were treated with a combination of THX and retraining/physiotherapy, whereas one was exclusively treated with retraining/physiotherapy in a training program targeting at improving WC. All patients had benefitted from treatment and were considered in a stable state of WC. Clinical data of the patients are displayed in Table 1.

**Table 1:** Overview of clinical data of examined WC patients

Gender	Age (in years)	Duration of FD (in years)	Handedness	Dystonic hand	Therapy
F	32.75	0.5	R	R	BT
F	66.75	0.75	R	R	BT
F	49.67	4	R	R	BT
F	56.25	10	R	R	Physical therapy, BT, Retraining
F	30.92	6	R	R	Physical therapy, BT, Retraining
F	33.33	12	R	B	BT, THX
F	26.67	14	R	R	Physical Training, Retraining
M	54.08	10	R	R	BT
M	44.33	12	R	R	BT
M	48.92	16	R	R	BT
M	42.83	13	R	R	Physical therapy, BT, Retraining
M	51.67	6	R	R	Physical Training, Retraining
M	69.67	19	R	R	Physical therapy, THX, Retraining
M	53	10	R	R	Physical therapy, THX, Retraining

Fourteen healthy controls without any neurological deficits were matched in gender, age and handedness. Mean age of the controls was 48.05 (SD: 13.88; range: 28.67 to 71.17).

Participants were asked to fill in a psychological questionnaire to assess the most important personality factors (the Big Five personality traits, NEO-FFI [24]). Furthermore, we assessed the impairment of daily tasks and symptoms of loss of control (Arm Dystonia Disability Scale, ADDS [25]). To distinguish temporary state anxiety from sustained trait anxiety, the State-Trait Anxiety Inventory, STAI [26], was used. Additionally, we asked to report accumulated lifetime practice of fine motor activities. Since all participants’ first language was German, standardized translations of the questionnaires were used [27].

Motor abilities were examined using 3 different test batteries. All participants began with the computer-assisted Motor Performance Test series, Vienna Test Series (https://www.schuhfried.com/test/MLS). This battery sub-divides into several fine motor manipulation tasks, including everyday life-like activities. Motor performance in handwriting and drawing was analyzed with the aid of a digitizing tablet, which has been used in a WC study by Zeuner and colleagues [28]. Finally we examined the grip force, as introduced by Hermsdörfer and colleagues [29].

Motor Performance Tests (MPT) and Grip Force Tasks (GFT) were carried out with both hands. Patients began these tests with their dystonic hand, and controls started with the hand corresponding to the affected hand of the respective matched patient. The Writing Task (WT) test was performed with the dominant hand exclusively.Patients started with the MPT, which requires the most steadiness, concentration and precision. Secondly, the WT was conducted, demanding less precision than the MP task. Furthermore, WT requires high activation of arm muscles, unlike MPT. Finally, GFT was conducted, predominantly registering grip force during lifting, holding, and moving an object, with less movement precision.Informed consent was obtained from all participants before study participation. The study was approved by the local ethics committee of the Hannover Medical University.

All variables obtained and analyzed in this study are displayed in Table 2.

**Table 2:** Overview of clinical data of examined WC patients

Biographical Variables		
Age		[years]
Sex		
Main Musical Instrument, Instrumental Group		strings, woodwind, none etc.
Musical Level		professional, amateur, non-musician
Level of Education		secondary modern school, grammar school final exams (comparable to UK A levels), vocational school, university degree
Previous Health Conditions		
Affected Hand in patients and respective hand in controls		
Handedness		
Lifetime Cumulative Fine Motor Activity: Handwriting, Keyboard Typing, Instrumental Music, Other, Summed; all: [yrs]		[years]
Psychological Questionnaires		
State Train Anxiety Inventory: State Anxiety, Trait Anxiety		
Arm Dystonia Disability Score		
NEO-FFI		
Motor Performance Tests		
Sub-Test	Measure	Right / Left / Bi-manual
Aiming	1) error number 2) error duration [ms] 3) total duration [s]	R, L, BR, BL
Steadiness	1) error number 2) duration [s]	R, L, BR, BL
Line Tracking	1) error number 2) error duration [ms]	R, L
Inserting of Long Pins	total duration [s]	R, L, BR, BL
Inserting of Short Pins	total duration [s]	R, L, BR, BL
Tapping	1) number of taps during first half of a 32 s recording 2) number of taps during second half 3) total number of taps	R, L, BR, BL
Writing Task		
Overall Writing Time		[s]
Frequency of the Written Trace		[s^−1^]
Mean Frequency of Up- and Downstrokes		[s^−1^]
Mean Axial Pressure on Pen		[N]
Writing Speed		[mm/s]
Length of Pen on Paper		[mm]
Doodling Circles		
Minimum Axial Pressure on Pen		[N]
Difference of Minimum Axial Pressure while Doodling and Mean Axial Pressure during Writing Task		[N]
Grip Force Tasks		
Maximum Grip Force (dystonic hand)		[N]
Maximum kinematic acceleration (dystonic hand)		[N]
Differential Load Force (dystonic hand)		[N]
Differential Grip Force (dystonic hand)		[N]
Mean Grip Force (dystonic hand)		[N]
Difference of mean Grip Force and Slip Force (dystonic hand)		[N]
Max Grip Force (non-dystonic hand)		[N]
Maximum kinematic acceleration (non-dystonic hand)		[N]
Differential Load Force (non-dystonic hand)		[N]
Differential Grip Force (non-dystonic hand)		[N]
Mean Grip Force (non-dystonic hand)		[N]
Difference of mean Grip Force and Slip Force (non-dystonic hand)		[N]
Grip force cyclic up-down Task (dystonic hand): min, max, mean and median		[N]
Grip force cyclic up-down Task (non-dystonic hand): min, max, mean and median		[N]

### Motor performance tests

**Fig. 1:**
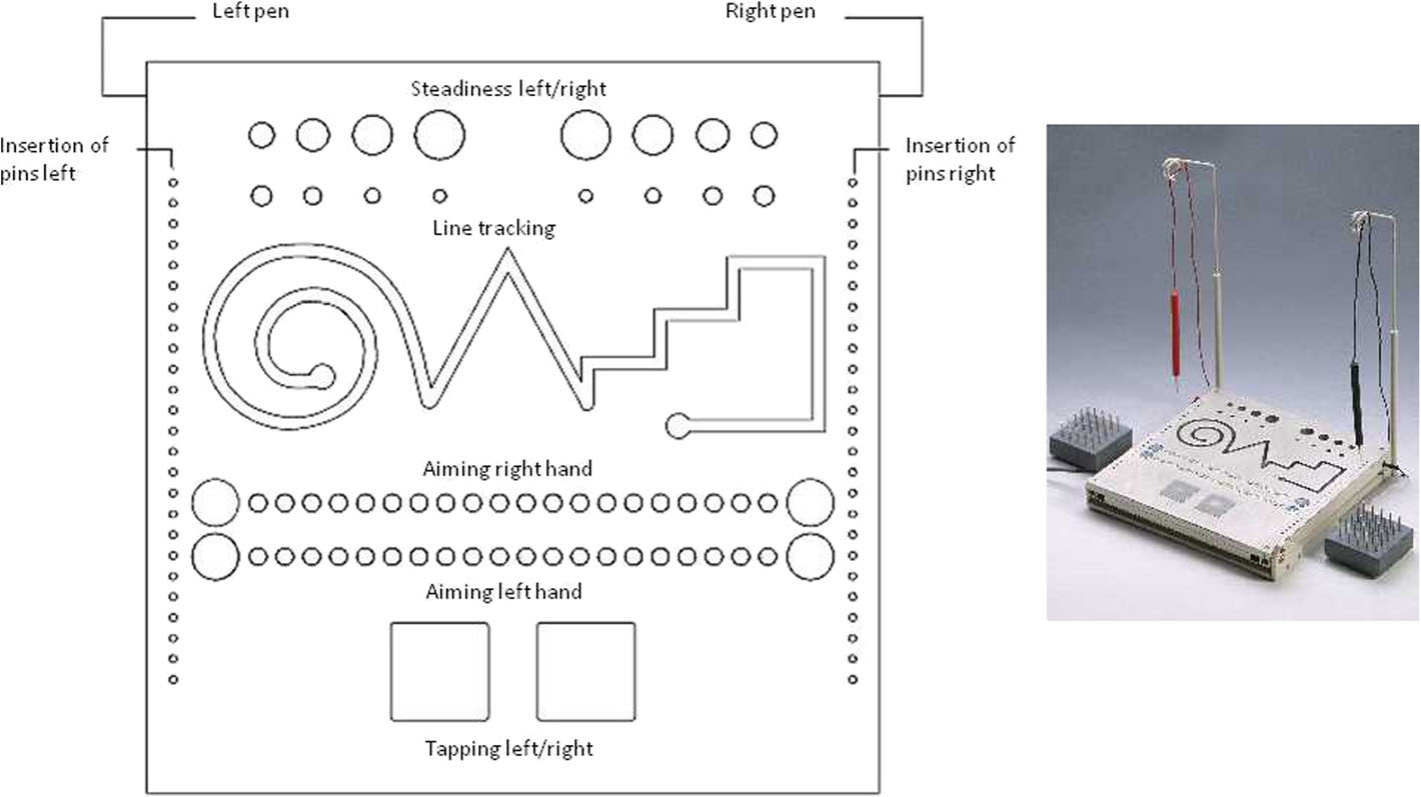
The MPT work panel (https://www.schuhfried.com/test/MLS), sketch kindly supplied by Schuhfried GmbH

To assess general fine motor skills, we used the MPT series (Schuhfried GmbH, Mödling, Austria, version 6.34.001). The work panel (see Fig. 1: the MPT work panel (https://www.schuhfried.com/test/MLS), W × H × D: 300 × 300 × 15 mm.) contained holes, grooves and contact surfaces. To perform most of the sub-tests, 2 metal rods (each 150 mm long, containing a 30 mm long contact pin) were used as a “pointing device” by the subjects. Nearly all tasks were performed with the dominant hand first and then with the non-dominant hand (see below for exceptions).

We applied the test form according to Schoppe & Hamster (https://www.schuhfried.com/test/MLS), which contains seventeen sub-tests. In the sub-test “Steadiness”, participants were required to steadily hold one of the metal rods with one hand in a hole (5 mm diameter), without touching the walls or the bottom, for a duration of 32 s. Number and duration of touches were recorded as errors in this sub-test, which can be considered as a test for tremor or involuntary shaking of the hand.

The “Line tracking” test required leading one of the metal rods through a 5 mm wide and 5 mm deep groove without touching the walls or the bottom. The required time and number of errors was recorded. This sub-test was only performed with the dominant hand.

The sub-test “Aiming” required the subjects to combine a fast vertical movement with a slower horizontal displacement of the limb by tapping successively on 20 adjacent metal circles with a metal rod as quickly as possible (circle diameter: 0.35 cm, midpoint-to-midpoint distance: 1.1 cm). Sliding the rod across the circles, as well as not touching a circle at all, was counted as an error. The number of errors and the required time were recorded.

For the “Tapping” sub-test, patients and controls tapped on a 50 mm × 50 mm metal surface with one of the metal rods as quickly as possible for 32 s. In this test, the number of taps was recorded separately for the first and second 16 s to account for possible fatigue effects, hypothesized to be higher in WC patients.

In the “Insertion” I and II sub-tests, subjects were asked to insert 25 pins, 50 mm and 10 mm long, respectively, into 25 target holes as quickly as possible. Longer pins were placed at a distance of 30 cm away from the work panel, shorter pins 10 cm away. The required time was measured.

### Writing tasks

Subjects wrote with a pressure-sensitive inking pen (WACOM Intuos3 pen, WACOM Europe, Krefeld, Germany) on a piece of ruled paper fixed on a digitizing tablet (WACOM Intuos3 A4 oversize with Grip Pen; WACOM Europe, Krefeld, Germany). Using this, we conducted a kinematic analysis of the writing to quantify the performed pressure, writing-speed and fluency. Registration and analysis of the data were done with CSWin Software (MedCom corp., Munich, Germany, version 2007). Resuming the writing tasks of Zeuner et al. [28], subjects performed 2 sub-tests.In the handwriting task, subjects wrote the sentence “Die Wellen schlagen hoch” (“The waves are surging high”) ten times in their normal handwriting. We used this sentence because of its facilitating sequences of letters which enable a quick and smooth writing style [28]. The test had to be performed within three minutes, which increased motivation and psychological pressure in patients and controls, in order to detect latent writing impairment. Data were analyzed exclusively from the word “Wellen” of the three first and three last sentences. We registered overall writing time [ms], number of pencil lifts, mean of axial pressure [N], mean frequency [s^−1^] of up- and downstrokes (vertical writing frequency), mean velocity [mm/s] and mean distance [cm] of the writing on the paper.The “Drawing Task” required subjects to draw as many superimposed circles with a diameter of about 2 cm as possible in 3 s, exerting as little vertical pressure on the pen tip as possible. We assumed that this task sensitively detects subtle changes in speed, smoothness and variability of successive movements [28], as circle drawing depends on the ability to accurately reproduce a typical movement pattern over time. In this sub-test, only the axial pressure was recorded. To assess the subjects’ ability of adaptation, the latter parameter was subtracted from the mean axial pressure obtained in the handwriting task.

### Grip force tasks

**Fig. 2:**
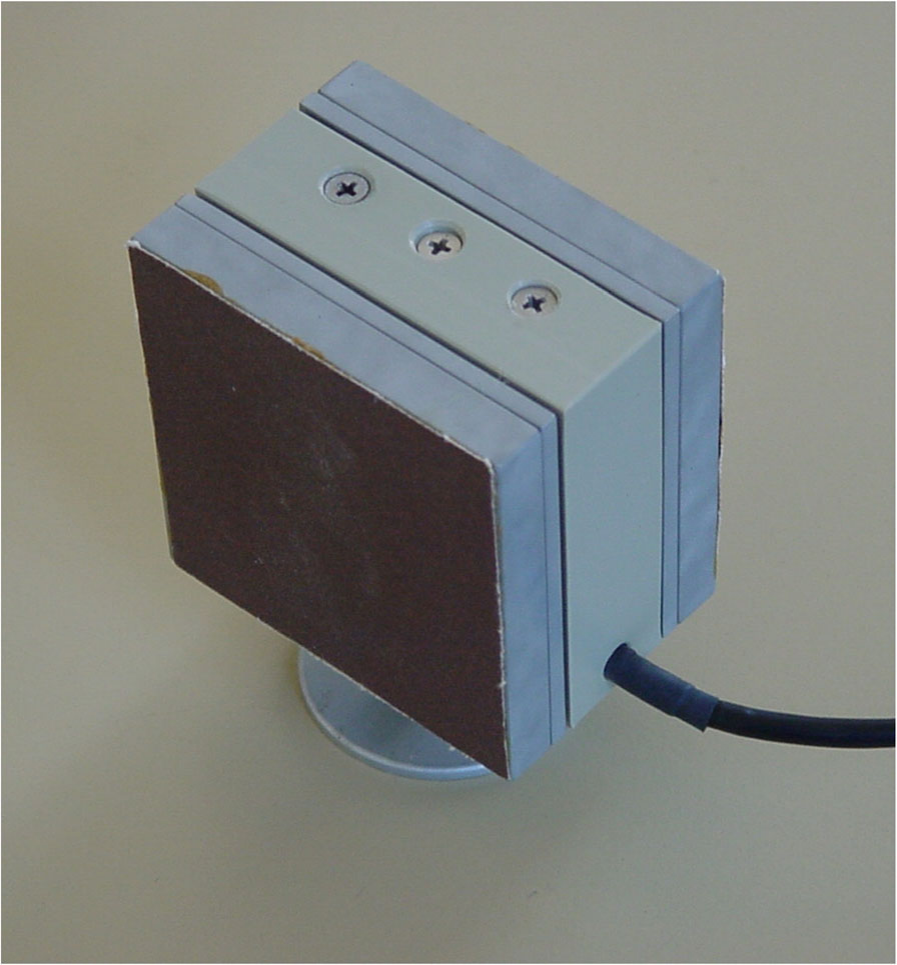
The Grip Force object

In these tasks, a metal block with integrated force and acceleration sensors and a total weight of 306.5 g (see Fig. 2: The Grip Force object. W × H × D: 65 × 65 × 50 mm) was used. Similar objects have been used in several studies by Hermsdörfer et al. [29]. We recorded grip force (0 – 80 N, accuracy ±0.1 N) and acceleration (50 m/s^2^, accuracy ±0.2 m/s^2^). After a/d-conversion (National Instruments USB-6009, sampling width: 14 bits, sampling rate: 100 Hz), the analysis of the signals was carried out with GF-Win software (Christian Marquardt, Munich, Germany).

In all tasks, subjects were instructed to only use thumb, index and middle finger (i.e. a tripod grip) to hold the object. Both sub-tests were carried out consecutively, first with the affected and then with the unaffected hand in patients, and with the respective matched hands in the control group.

### Lifting task

Subjects sat on a chair in front of a small object, holding the upper arm parallel to the trunk and the forearm unsupported to the front. When an acoustic cue sounded, the object was lifted approximately 4 cm above the table, held there for 5 s and then placed back on the desk [29]. Patients and controls repeated this task 30 times (15 trials per hand) with intervals of 2 s between trials. Patients began with their dystonic hand, the matched controls with their respective hand.

### Slipping task

We applied a procedure to evaluate the minimal grip force needed to hold the object, originally introduced by Johansson and Westling [30]. Subjects were instructed to hold the object and reduce the grip force as slowly as possible until the object slipped from their fingers. This was repeated twice, resulting in a total of three slip force trials. Again, patients began with their dystonic hand.

During the lifting and slipping tasks, we measured various data, which were collected for all 30 trials. We recorded GFMax [N] (maximal force after the object had been lifted up), AccMax [m/s^2^] (kinematic acceleration during lifting phase of the object), and MeanGF [N] (during the static phase). In addition to this, we calculated the ratio GFMax/LFMax (sensitive measurement for the efficiency of grip force in relation to the lifting-induced load) and TLift-off [ms] (time until object was lifted off). For the slipping task, we registered the mean slip force (GFSlip [N]) for all three trials and also calculated the difference of MeanGF and GFSlip to obtain a sensitive value for the subject’s ability to adapt to the weight while grasping and lifting an object.

### Cyclic movement task

Patients were instructed to lift the object and to move it up and down for a period of 15 s with an amplitude of approximately 30 cm and a tempo corresponding to 65BPM as paced by a metronome, beginning with their dystonic hand. Matched controls started with their respective hand. Every five trials hands were switched. To analyze the performance of this task, minimum, maximum, mean and median grip forces of the first 9000 ms of all trials of the cyclic up-down movements were taken into account.

### Statistics

Psychological questionnaire data were compared between groups using Wilcoxon’s signed rank test. To identify differences in motor performance between WC patients and healthy controls, the applied motor test battery scores of dystonic hands and the corresponding hands of the matched controls were compared. For investigating task-specificity of WC, variables were grouped test battery-wise and subjected to a random forest ensemble supervised learning algorithm [31]. Random forests are large collections or ensembles of many classification trees (10,000 in this study). A single classification tree partitions a data set recursively by locally assessing, at each node, which predictor or variable distinguishes best between patients and controls. The resulting two daughter nodes then exhibit a maximally reduced impurity with respect to the response variable. An error rate is obtained by randomly assigning observations to learning and testing subsets, respectively, followed by cross-validation after learning has taken place. This randomized pre-partitioning renders the results of single trees unreliable, as no two successive runs on the same data set will yield the same result. This shortcoming is resolved by adding randomness by growing many such trees (thus a random forest) on subsampled subsets of the data (average subsample size: 0.632 * *n*, with *n* the number of total observations [32]. In each tree and at each node, the best splitting variable from a random subset of all predictors is found. The forest’s majority vote is then used to classify the observations as either patient or control. A conditional permutation importance measure is also provided, allowing an estimation of the relative influence of all predictors on the response variable [33]. For classification, the function “cforest” from the R package “party” was used [34].

This method provides tables listing the number of cases in which the algorithm correctly and incorrectly classified patients and controls. These confusion matrices were subjected to Pearson’s Chi^2^-test.

The advantage of using this data-driven classification rather than classical hypothesis-driven methods, like logistic regression, is its ability to determine complex interactions even in small *n* large *p* problems with more predictors than observations, where methods from the General Linear Model framework would fail.

To assess the degree of the “focal” character of WC, forests on Motor Performance and grip force data were examined separately for the left and right hand.

To determine which dependent variables contributed most to differentiation between patients and controls, the entire set of dependent variables (with a few exceptions) was used to grow a forest. To avoid trivial results, we excluded all WT and ADDS data before training. This resulted in a data set with 96 variables. As the hand movement in the line tracking task in the MPT battery vaguely resembles writing movements – though from right to left instead from left to right – we additionally excluded those data before running the classification algorithm a second time on then 88 dependent variables.

The level of significance for all tests was set to α = 0.05. All statistical computations were done in R, version 3.0.1 [34].

## Results

### Task-specificity

**Fig. 3:**
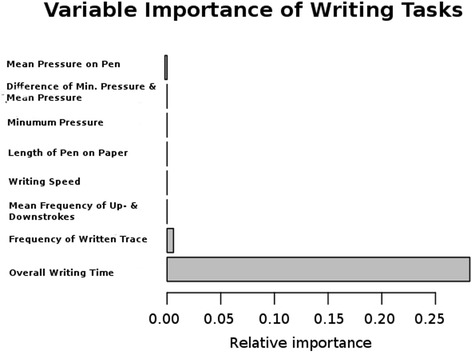
Relative variable importance to maximize node purity in Writing Task data subjected to a random forest. Unsurprisingly, overall writing time was largest in the patient group

Thirty-four randomly selected variables from the MPT data were used at each node to find a split. Nine of the 14 patients (64%) and 10 of 14 controls (71%) were correctly classified by the random forest (χ^2^ = 2.297, df = 1, *p* > 0.05). The WT data (8 variables used) yielded a correct classification rate of 100% and 86% for patients and controls, respectively (χ^2^ = 17.65, df = 1, *p* < 0.05). The variable importance measure showed the total duration of writing and the vertical writing frequency of the pen to be the most important variables used to split the data set successfully into patients and controls (see Fig. 3:Variable Importance of Writing Tasks.). Grip force data (16 variables) returned rates of 43% and 57% (χ^2^ = 0, df = 1, *p >* 0.05).

### Focal nature of Dystonia

Correct classification rates on the basis of MPT sub-tests performed with the right hand scored 71% and 57% in patients and controls, respectively (χ^2^ = 1.312, df = 1, *p* > 0.05). Those performed with the left reached 43% and 57% (χ^2^ = 0, df = 1, *p* > 0.05). Data in sub-tests performed bi-manually were analyzed independently for both hands. The correct classification rate for the bi-manual right hand data amounted to 64% and 57% (χ^2^ = 1.292, df = 1, *p* > 0.05), while those for bi-manual left hand data reached 57% and 50% (χ^2^ = 0.1436, df = 1, *p* > 0.05). Right hand grip force data amounted to 36% and 43% (χ^2^ = 1.292, df = 1, *p* > 0.05), those from the left hand reached 64% and 57% (χ^2^ = 1.292, df = 1, *p* > 0.05).

### Psychological questionnaires

The State Anxiety and the Trait Anxiety Inventory (STA-I) scores differed between both groups: Median difference [95% CI] -5.0 [−10.0, −1.5], *V* = 13.5, *p* = 0.016, and −6.5 [−11.5, −1], *V* = 15, *p* = 0.02, respectively. WC patients had more state and trait anxiety as compared to healthy subjects.

Our data did not provide evidence that groups differed in the Big-Five (Neuroticism: −5 [−11, 0.9], *V* = 25, *p* = 0.09; Extraversion: 3.5 [−2.5, 10.0], *V* = 72.5, *p* = 0.22; Openness: 1.5 [−7.0, 9], *V* = 56, *p* = 0.85; Agreeableness: 1.0 [−4.0, 3.5], *V* = 27, *p* = 0.62; Conscientiousness: −0.5 [−4.9, 3.5], *V* = 41, *p* = 0.78.

### Data exploration

To explore the features separating patients and controls, a random forest was grown over the entire data set, including biographical data and the STAI questionnaire, amounting to 96 dependent variables. From these 96, 72 randomly selected variables were used at each node to choose the best split point from. The resulting classification rates were 100% and 86% for patients and controls, respectively (χ^2^ = 17.65, df = 1, *p* < 0.05). Not very surprisingly given the results in the Task-Specificity section, the total duration of writing and the vertical writing frequency of the pen significantly contributed to the splitting of the data set. Their predictive power was so pervasive that the classification rates and the result of the χ^2^ test were identical to those in the above section. After the exclusion of WT data (66 randomly chosen variables from a total of 88), rates went down to 57% and 71% (χ^2^ = 1.312, df = 1, *p* > 0.05).

## Discussion

### Task-specificity

Apart from the writing task, our extensive fine motor testing battery failed to detect any differences between WC and healthy controls. Although WC has previously been frequently described as focal and task-specific dystonia, other researchers found signs of reduced range of finger motion, or spread to secondary motor disturbances [9, 13]. It has been discussed whether, among these secondary motor disturbances, primarily movements similar to the major affected task could be affected [17]. This would confirm the notion that WC is more movement- than task-specific and would therefore have implications for therapies, e. g. retraining [35]. Here, we do not have any evidence that tasks closely related to writing, such as the drawing task or the steadiness task in the Vienna testing battery, displayed any differences. Furthermore, increased grip force, which is a hallmark of WC, was not present in the grip force and slipping tests. In the WT, only total duration of writing and vertical writing frequency of the pen explained most of the variance separating patients and controls, showing that patients had more difficulties performing the WT. We therefore explain the lack of further variables suitable to classify patients and controls in the MPT by the absence of spread of motor symptoms in our population of WC patients. This implies that our sample patients exhibit a task-specific and focal WC, thus supporting the results of Schneider et al. [19] With respect to grip-force, Nowak et al. claimed that increased grip-force levels are learned and context specific phenomena [36], and most probably subject to the sensory trick phenomena when tested in a short-time paradigm [37].

Even though we recruited a cohort with stable patients, many factors may have contributed to a possible heterogeneity of our patient group. Eight of 14 patients were amateur musicians and, though none were diagnosed with MD, difficulties in musician’s performance had been reported by 5 patients. It should be mentioned that in Germany, about 18% of the population play a musical instrument, and even more in the WC population (mainly school-teachers, lawyers and doctors). We cannot exclude that musical performance as an environmental factor may have contributed to the triggering of WC [37, 38]. However, these amateurs did not practice more than 3 h a week and fine motor workload was probably much more increased by computer typing and handling smart phones. Moreover, since 12 of 14 patients in our study were diagnosed with WC more than 4 years ago, adaptation of the writing style over time may have occurred. Furthermore, as eight of 14 patients were treated with combination of at least 2 different therapeutic options, less motor impairment within our patient group in comparison to other severely affected cohorts must be considered.

Ideally, future studies should examine untreated WC patients with a shorter history of FD. Pen grip force should be additionally assessed, although we expect that this will be increased, as writing time was slowed in our study.

### Psychological profile

Given that psychological symptoms have been demonstrated to be present in MD [20], it is widely discussed whether or not non-motor symptoms are also common in WC [21], demanding reconsideration of etiological factors and additional therapeutic approaches, for example behavioral training. In this context, it is noteworthy that our data suggest that at least state and trait anxiety seem to be elevated in WC patients as compared to healthy matched controls, whereas the NEO-FFI subscales did not provide evidence that the patients differ from the controls.

Classification of patients and controls using personality factors and anxiety traits was unsuccessful, thus failing to provide support for a possible connection between psychological and motor symptoms. This is in contrast to results of Ioannou et al., who could show that a majority of MD patients suffer from psychological symptoms, which might even trigger dystonic symptoms [20]. Such a clear connection between MD and certain personality traits [39] may be partially due to the impact of professional pressures in musicians, which is obviously less pronounced in WC patients. Moreover, the role of overuse and prolonged practice as triggering factors in WC seem to only play a minor role [21]. These results could, if confirmed with a new data set, support the notion that there are etiological differences within subtypes of FD, as the latter have been already discussed [13, 40].

However, as Enders et al. showed psychiatric comorbidities of FD [41], it is important to not disregard psychodynamic developments, psychoreactive aspects (especially the patients’ experience of FD), as well as influences of the disorder concerning everyday activities and life quality [42, 43]. This can be achieved in future studies by using psychological and psychiatric case reports, and should be accomplished by obtaining thoroughly detailed anamnesis.

## Conclusion

Only total duration of writing and vertical writing frequency of the pen correctly separated patients and controls, corroborating that patients had more difficulties performing the WT, indicating task-specificity within our patient group. We suggest that similar research should be performed on larger samples of simple WC patients in early stages of impairment without reported clinical secondary motor disturbances to verify our findings, using logistic regression techniques.

Our manuscript does not contain any individual persons data.

## Abbreviations

ADDS: Arm Dystonia Disability Scale
GF: Grip Force
GFT: Grip Force Task
MD: Musician’s Dystonia
MPT: Motor Performance Test
NeoFFI: Neo Five-Factor Inventory
STAI: State-Trait Anxiety Inventory
WC: Writer’s cramp
WT: Writing Task
